# Selenium and Copper as Biomarkers for Pulmonary Arterial Hypertension in Systemic Sclerosis

**DOI:** 10.3390/nu12061894

**Published:** 2020-06-25

**Authors:** Qian Sun, Julian Hackler, Julia Hilger, Hans Gluschke, Aldina Muric, Szandor Simmons, Lutz Schomburg, Elise Siegert

**Affiliations:** 1 Institute for Experimental Endocrinology, Charité-Universitätsmedizin Berlin, corporate member of Freie Universität Berlin, Humboldt-Universität zu Berlin, and Berlin Institute of Health, D-13353 Berlin, Germany; qian.sun@charite.de (Q.S.); julian.hackler@charite.de (J.H.); hans.gluschke@charite.de (H.G.); aldina.muric@fu-berlin.de (A.M.); 2 Department of Rheumatology and Clinical Immunology, Charité-Universitätsmedizin Berlin, corporate member of Freie Universität Berlin, Humboldt-Universität zu Berlin, and Berlin Institute of Health, D-10117 Berlin, Germany; julia.hilger@charite.de; 3 Institute of Physiology, Charité-Universitätsmedizin Berlin, corporate member of Freie Universität Berlin, Humboldt-Universität zu Berlin, and Berlin Institute of Health, D-10117 Berlin, Germany; szandor.simmons@charite.de

**Keywords:** trace element, inflammation, selenoprotein P, ceruloplasmin, glutathione peroxidase

## Abstract

Circulating selenoprotein P (SELENOP) constitutes an established biomarker of Se status. SELENOP concentrations are reduced in inflammation and severe disease. Recently, elevated SELENOP levels have been suggested as diagnostic marker and therapeutic target in pulmonary arterial hypertension (PAH). We decided to re-evaluate this hypothesis. A group of healthy controls (*n* = 30) was compared with patients suffering from systemic sclerosis (SSc, *n* = 66), one third with SSc-related PAH. Serum was analysed for trace elements and protein biomarkers, namely SELENOP, glutathione peroxidase 3 (GPx3) and ceruloplasmin (CP). Compared to controls, patients with SSc-related PAH displayed reduced serum Se (91 ± 2 vs. 68 ± 2 µg/L) and SELENOP concentrations (3.7 ± 0.8 vs. 2.7 ± 0.9 mg/L), along with lower GPx3 activity (278 ± 40 vs. 231 ± 54 U/L). All three biomarkers of Se status were particularly low in patients with skin involvement. Serum Cu was not different between the groups, but patients with SSc-related PAH showed elevated ratios of Cu/Se and CP/SELENOP as compared to controls. Our data indicate that patients with SSc-related PAH are characterized by reduced Se status in combination with elevated CP, in line with other inflammatory diseases. Further analyses are needed to verify the diagnostic value of these TE-related biomarkers in PAH.

## 1. Introduction

Some trace elements (TE) are essential for human health, and deficiencies are associated with disease risk and a more severe disease course. The requirements vary with age [1] and, especially, the immune system depends on a balanced and sufficiently high TE supply [2]. The essential TE selenium (Se) is unique within the group of micronutrients, as it is part of the amino acid selenocysteine (Sec) and as such becomes directly incorporated into selenoproteins during translation [3]. Selenoproteins are an instructive example for the importance of TE supply, as their biosynthesis depends directly on the dietary intake of Se that differs strongly across the world [4]. Accordingly, some populations are considered as being sufficiently supplied by their regular diet, e.g., subjects living in North America, whereas Se availability in large parts of Europe, Asia and Africa is marginal and even tends to decline [5]. Certain health risks are directly related to Se deficiency, e.g., viral infections as well as Kashin-Beck and Keshan disease in the Se-poor areas of Asia, where targeted Se supplementation is a successful preventive measure [6,7]. Several biomarkers for detecting Se deficiency and monitoring successful supplementation effects have been evaluated [8]. Among the different candidates, three parameters emerged as the most informative ones, i.e., enzymatic activity of the secreted glutathione peroxidase (GPx3) in serum or plasma, concentration of total serum or plasma Se, and the expression level of the circulating Se transporter selenoprotein P (SELENOP) [9,10,11]. These biomarkers correlate over a wide range of Se intakes, since they are not independent from each other and ultimately rely on Se availability and selenoprotein biosynthesis. Accordingly, supplemental Se intake is reflected in a parallel increase in all three biomarkers in clinical studies with marginally supplied subjects [9]. Inversely, genetic defects in selenoprotein biosynthesis or severe disease negatively affect all three Se status biomarkers [12]. As micronutrient, Se is taken up by the diet in different forms undergoing a variety of biochemical conversions [13,14], resulting in compound-specific bio-availabilities and toxicities, respectively [15,16].

Besides low Se intake and inherited defects, many common diseases are also related to a suboptimal Se status in a more complex manner, where sometimes cause and consequences are difficult to disentangle [17,18,19]. A Se deficit is known to predispose to, e.g., liver or colorectal cancer [20,21], to thyroid [22,23] and cardiovascular disease [24,25], or to survival odds in sepsis [26], after poly-trauma [27], in breast [28], lung [29] or renal cancer [30]. Notably, the stringent interrelation between Se deficit and mortality risk may be confined to subjects residing in areas with low baseline Se supply, where a declining Se status may reach a certain essential minimal threshold more rapidly than under ample Se supply. This hypothesis has received support by a recent analysis of cure rates from SARS-CoV-2-related COVID-19 in relation to baseline Se status in different areas of China, and is discussed as of potential relevance for COVID infection, disease spread and course [31,32].

Inflammation, hypoxia, pro-inflammatory cytokines and other stress signals are among the parameters accelerating and aggravating a Se status decline [33,34,35]. Hence, we hypothesized that in a disease such as systemic sclerosis (SSc), which is characterized by inflammation, vasculopathy and fibrosis with resulting hypoxia, patients may present with low Se status. Furthermore, in view of the fact that SSc-related pulmonary arterial hypertension (SSc-PAH) is the final presentation of progressive pulmonary vasculopathy, we assume that this condition may be associated with particularly depleted Se status, i.e., depressed values of total Se and SELENOP concentrations. A similar association may also apply to SSc-related skin changes, as they are associated with declining lung function [36], hypoxia and mortality risk in SSc [37]. Given the severity of the disease and the urgent need for an improved diagnosis and helpful indicators of disease risk and progression, we decided to re-evaluate different biomarkers of TE status in patients with SSc and SSc-PAH in comparison to healthy controls.

## 2. Materials and Methods

### 2.1. Study Design

A cross-sectional study of healthy controls and patients with SSc fulfilling the American College of Rheumatology (ACR)/European League Against Rheumatism (EULAR) 2013 classification criteria [38] was conducted. Around one third of the SSc patients were suffering from SSc-PAH (Table 1). Diagnosis of SSc-PAH was based on the 2015 European Society of Cardiology (ESC)/ European Respiratory Society (ERS) guidelines for the diagnosis and treatment of pulmonary hypertension [39]. All study subjects enrolled into the analysis had provided a written informed consent form and their samples were deposited in a local biobank. The study was conducted in accordance with the Declaration of Helsinki. Approval was granted by the Board of Ethics of Universität zu Köln (#04-037), and Charité Medical School Berlin (#EA1/178/17 and #864-14). The samples had been stored at −80 °C until analysis. All measurements were conducted blinded to any clinical information.

### 2.2. Trace Element Analysis

Concentrations of serum TE were determined by total reflection X-ray fluorescence (TXRF) analysis using a benchtop TXRF analyzer (S2 Picofox, Bruker Nano GmbH, Berlin, Germany), essentially as described previously [21]. Briefly, 10 µL of serum sample was diluted with an equal volume of a gallium standard (1000 µg/L), 8 µL of the dilution was applied to a polished quartz glass slide and samples were dried overnight. Seronorm serum standard (Sero AS, Billingstad, Norway) served as control, and the Se concentrations determined were within the specified range of the standard and linear, on dilutions in the range of 1:3, 1:10 and 1:20. The inter- and intra-assay CV was determined to be below 10% in the concentration range of 50–150 µg Se/L serum.

### 2.3. SELENOP and CP Quantification by ELISA, and Analysis of Serum GPx3 Acticity

Serum SELENOP concentrations were measured by sandwich ELISA using a validated commercial SELENOP-specific ELISA (selenOtest^TM^, selenOmed GmbH, Berlin, Germany), essentially as described [40]. Briefly, serum samples of 5 µL were diluted 1:33 and applied to pre-coated 96-well plates. Standards and calibrators were included into each assay run for quality control.

Serum CP concentrations were determined by a validated non-competitive ELISA as described recently [41]. Briefly, serum samples were pre-diluted 1:300 in sample buffer, and 50 μL of diluted sample were incubated on pre-coated sandwich ELISA plates for 30 min at room temperature. After several wash steps, the plates were incubated with detection antibody conjugated with horseradish peroxidase for 30 min. Following further wash steps, the enzymatic detection reaction was started by adding 100 μL of 3,3′5,5′-Tetramethylbenzidine (TMB) substrate and terminated by adding an equal volume of sulfuric acid. Spectrophotometric readout at 450 nm was recorded by a microplate reader (Tecan Group AG).

GPx3 activity was determined by a coupled enzymatic test procedure monitoring reduced nicotinamide adenine dinucleotide phosphate (NADPH) consumption at 340 nm [42]. Briefly, serum samples of 5 µL were applied to 96-well plates. After adding 200 µL of a test mix including 1 mM NaN_3_, 3.4 mM reduced glutathione, 0.3 U/mL glutathione reductase and 0.27 mg/mL NADPH, the test was started by 10 µL of 0.00375% hydrogen peroxide. The decrease in NADPH absorbance per minute measured at 340 nm as readout is proportional to the GPx3 activity in the sample. A constant serum sample was included into each assay run for quality control. The inter- and intra-assay CV was determined to be below 15%.

### 2.4. Statistical Analysis

Statistical analysis was performed with SPSS Statistics ^®^ (version 25, IBM, Chicago, IL, USA) and GraphPad Prism (Version 7, GraphPad Software Inc., San Diego, CA, USA), respectively. Normal distribution of values was tested by the Shapiro-Wilk test. Comparisons between two groups were conducted by unpaired t test, and for not-normally distributed variables with Mann-Whitney test. Comparisons of the characteristics between more than two groups were conducted with ANOVA and Dunn’s multiple comparisons test, and for not-normally distributed variables with the Kruskal-Wallis test. Correlations were tested by Pearson’s correlation analysis and for not-normally distributed variables by Spearman’s correlation test. All statistical tests were two-sided and *p*-values < 0.05 were considered statistically significant; * *p* < 0.05, ** *p* < 0.01, *** *p* < 0.001, and **** *p* < 0.0001.

## 3. Results

### 3.1. Patient Data

A total of 66 SSc patients met the ACR/EULAR 2013 classification criteria, and 30 healthy controls (HC) were included into the analysis. The study population showed the expected female predominance and a mean age range typical for SSc (Table 1).

### 3.2. Selenium (Se) and Copper (Cu) Status Assessments

**Figure 1:**
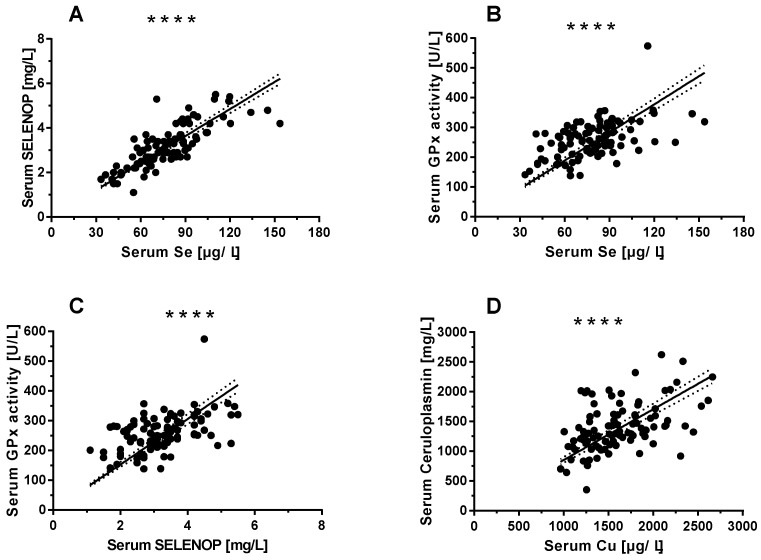
Selenium (Se) and Copper (Cu) status assessment from serum of the full study cohort. The concentrations of biomarkers of Se status correlate significantly in the serum samples analysed, and positive and tight correlations are observed for (**A**) selenoprotein P (SELENOP) and total Se (*n* = 96 pairs of data, *r* = 0.792, *p* < 0.0001), (**B**) GPx3 activity and total Se (*n* = 96, *r* = 0.491, *p* < 0.0001), as well as (**C**) GPx3 activity and SELENOP (*n* = 96, *r* = 0.470, *p* < 0.0001). (**D**) Similarly, both biomarkers of Cu, i.e., ceruloplasmin (CP) and total Cu concentrations show a positive and tight correlation across the full range of values in the complete cohort of serum samples (*n* = 96, *r* = 0.475, *p* < 0.0001). Correlations were analysed by Spearman’s correlation test. *p*-values < 0.05 were considered statistically significant; **** indicates *p* < 0.0001.

Serum Se status was evaluated by three complementary biomarkers, i.e., total serum Se (Figure 1A) and SELENOP concentrations (Figure 1B), we well as GPx3 activity (Figure 1C). The three biomarkers of Se status showed significant and linear correlations over the full range of data, indicating a high quality of the samples. Cu status was evaluated by two complementary biomarkers, i.e., total serum Cu and CP concentrations. The assessment of the full cohort of samples indicates a linear correlation of both Cu biomarkers verifying the integrity of the samples and quality of analysis (Figure 1D).

### 3.3. Comparison of TE Status in SSc Patients with and without PAH Versus Healthy Controls

**Figure 2:**
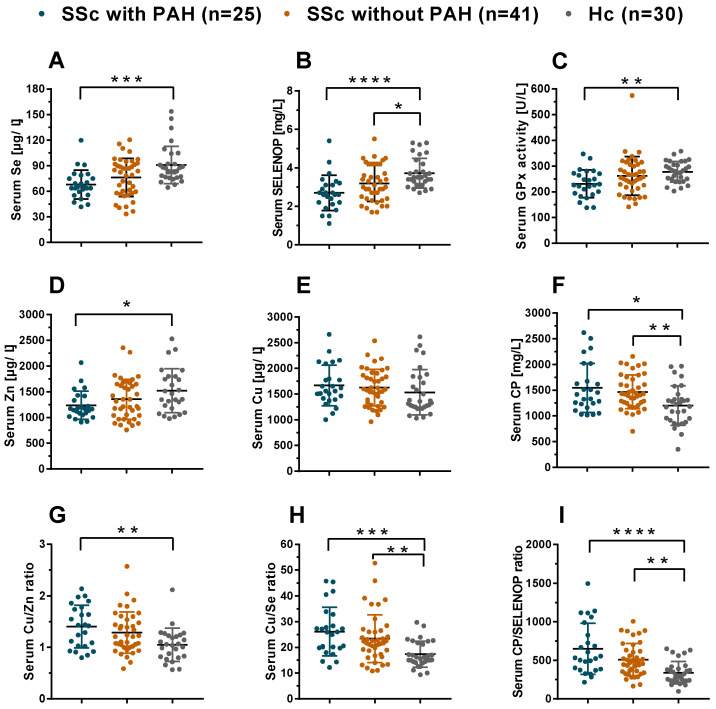
Comparison of TE status and composite biomarkers of TE in SSc patients versus controls. Patients suffering from SSc-PAH display reduced serum Se and Zn status as compared to healthy controls (HC). Differences are observed for (**A**) total Se and, (**B**) serum SELENOP concentrations, as well as (**C**) serum GPx3 activity and (**D**) total serum zinc (Zn) concentrations. In comparison, (**E**) total serum Cu concentrations do not differ between the groups. Concentration of the Cu transporter CP is elevated in SSc-PAH patients as compared to HC. Similar differences are detected for (**F**) serum ceruloplasmin (CP) concentrations, (**G**) serum Cu/Zn ratio, (**H**) serum Cu/Se ratio, and (**I**) serum CP/SELENOP ratio. Comparisons were analysed by Kruskal-Wallis statistics and Dunn’s multiple comparisons test. All tests were two-sided and *p*-values < 0.05 were considered statistically significant; * indicates *p* < 0.05, ** indicates *p* < 0.01, *** indicates *p* < 0.001 and **** indicates *p* < 0.0001.

The SSc patients displayed disease-specific differences in the TE status biomarkers in comparison to HC (Figure 2). To further subdivide the analyses, the group of SSc patients was differentiated according to presence or absence of PAH. A gradual decrease in Se status from HC to patients with SSc and then with SSc-PAH was observed. The group of SSc-PAH patients displayed the lowest Se status, i.e., significantly reduced concentrations of Se (Figure 2A), and SELENOP (Figure 2B), as well as reduced GPx3 activity (Figure 2C). Notably, the average levels of all three Se status biomarkers were consistently lower in patients with SSc-PAH than in HC. In comparison to established reference ranges from literature [21,43], the SSc patients displayed a relative Se deficit (Table S1); the prevalence of Se deficiency (SSc-PAH vs. SSc vs. controls) was 16.0% vs. 14.6% vs. 0% as judged by serum Se, 64.0% vs. 41.5% vs. 10% as judged by serum SELENOP, and 28.0% vs. 19.5% vs. 0% as judged by GPX3 activity, respectively.

Besides Se, the biomarkers of Cu and Zn status were also analyzed. Total serum Zn concentrations were relatively low in SSc-PAH as compared to HC (Figure 2D), whereas total serum Cu concentrations were not different between the groups (Figure 2E). The protein biomarker of Cu status, i.e., ceruloplasmin (CP), was elevated in SSc, both in patients with and in patients without PAH, as compared to HC (Figure 2F). TE status was also analysed by composite biomarkers, that may provide more sensitive information. To this end, ratios were built between serum TE and the protein biomarkers, respectively. In this analysis, consistently elevated values in the patients are observed for the Cu/Zn ratio (Figure 2G), Cu/Se ratio (Figure 2H) and CP/SELENOP ratio (Figure 2I), respectively.

**Figure 3:**
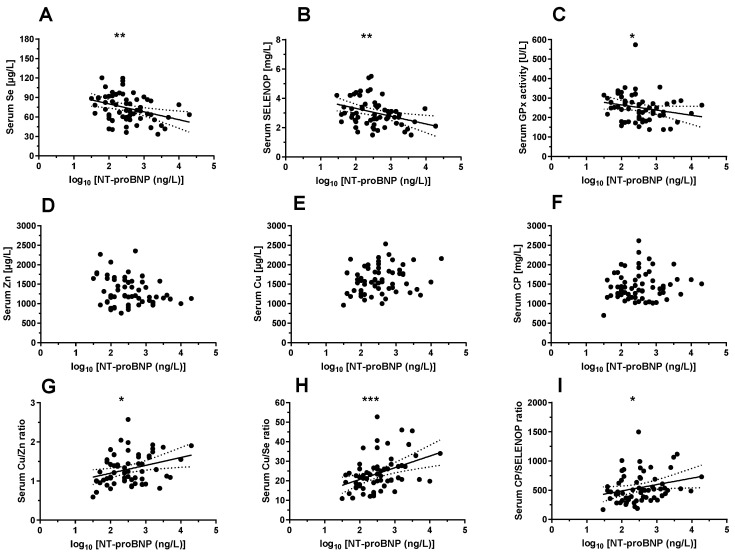
Biomarkers of TE status in relation to normalized NT-proBNP level. A comparison of the biomarkers of TE status to an established biomarker of SSc-PAH reveals significant interactions for most of the TE parameters analysed. Among the biomarkers of Se status, (**A**) serum Se (n = 63, r = −0.351, *p* = 0.005) and, (**B**) SELENOP concentrations (n = 63, r = −0.344, *p* = 0.006), as well as (**C**) serum GPx3 activities (n = 63, r = −0.318, *p* = 0.011) show a significant and consistent negative correlation with N-terminal pro-B-type natriuretic peptide (NTproBNP). No significant interaction was detected for (**D**) total serum Zn (*p* = 0.125), (**E**) total serum Cu (*p* = 0.083), or (**F**) serum CP concentrations (*p* = 0.634). Some composite biomarkers of TE status were positively related with NTproBNP, as seen in an analysis of the (**G**) Cu/Zn ratio (r = 0.316, *p* = 0.014), (**H**) Cu/Se ratio (r = 0.423, *p* = 0.0005), and (**I**) CP/SELENOP ratio (r = −0.293, *p* = 0.020). Correlations between parameters (n = 63) were tested by Pearson’s correlation analysis, and for not-normally distributed variables by Spearman’s correlation test. *p*-values <0.05 were considered statistically significant; * indicates *p* < 0.05, ** indicates *p* < 0.01, and *** indicates *p* < 0.001.

The N-terminal pro-B-type natriuretic peptide (NTproBNP) has been shown as valuable biomarker for heart failure, and NTproBNP concentrations are elevated in patients suffering from SSc-PAH [44]. A direct comparison of the TE concentrations, the protein biomarkers and their ratios with NTproBNP supports the consistency of the data and the potential usefulness of TE-based biomarkers for improving SSc-PAH diagnosis (Figure 3).

### 3.4. Analysis of TE Biomarkers in SSc Patients in Relation to Skin Involvement

**Figure 4:**
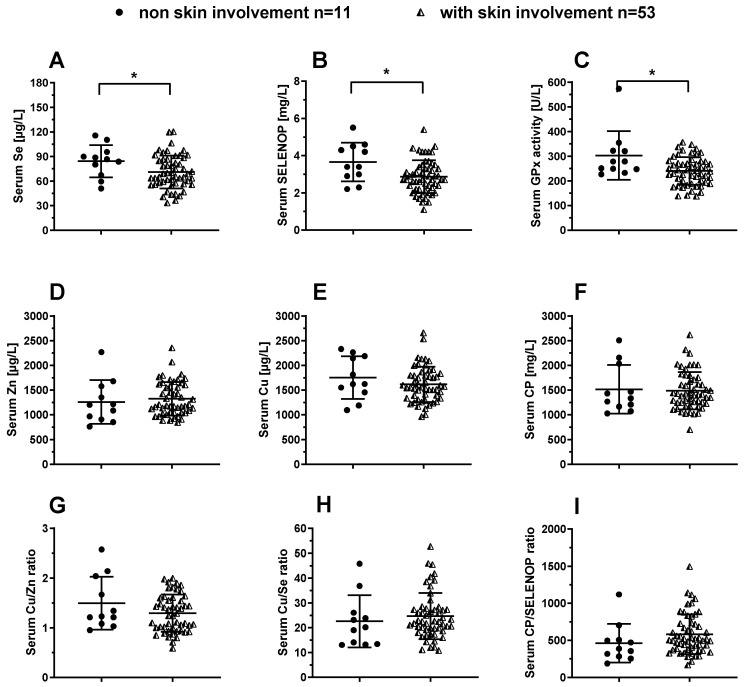
Comparison of TE biomarkers in SSc patients in relation to skin involvement. The average Se status is reduced in SSc patients with skin involvement as compared to the patients without skin involvement. The SSc patients with skin involvement display relatively low (**A**) total serum Se, and (**B**) SELENOP concentrations, as well as (**C**) reduced serum GPx3 activity, in comparison to SSc patients without skin involvement. No difference between the groups are observed for (**D**) total serum zinc (Zn), and (**E**) total serum Cu concentrations. Similarly, (**F**) serum ceruloplasmin (CP) concentrations, (**G**) the Cu/Zn, (**H**) Cu/Se, and (**I**) CP/SELENOP ratio do not differ between the groups. Comparisons between two groups were conducted by unpaired t test, and for not-normally distributed variables with Mann Whitney test. All tests were two-sided and *p*-values < 0.05 were considered statistically significant; * indicates *p* < 0.05.

Skin involvement in SSc is associated with severity of disease. The cohort of patients was sub-divided into two groups according to skin involvement as assessed by the modified Rodnan skin score (mRSS). The group of SSc patients with skin involvement displayed relatively low values for total serum Se (Figure 4A), and SELENOP concentrations (Figure 4B), as well as for GPx3 activity (Figure 4C). Notably, all three Se status biomarkers were consistently lower in SSc patients with skin involvement as compared to SSc patients without skin involvement. In comparison to serum Se status, there were no differences in total serum Zn (Figure 4D), Cu (Figure 4E) or CP (Figure 4F) concentrations, as well as no differences in the Cu/Zn (Figure 4G), Cu/Se (Figure 4H) or CP/SELENOP ratio (Figure 4I). In an analysis according to the three major forms of cutaneous involvement (limited, diffused or sine scleroderma), no significant differences in any of the markers were observed between the groups.

## 4. Discussion

In this manuscript, we report the TE status of SSc patients with or without PAH in comparison to HC, as assessed by a number of complementary biomarkers. The data indicate several significant differences between the groups that are compatible with the inflammatory nature of SSc, and particularly pronounced alterations are recorded in relation to PAH. The validity of the data is supported by several findings, e.g., by the tight and linear correlations of all three biomarkers of Se status over the full concentration ranges, and by their consistent relation to SSc and PAH severity. The latter was assessed by the presence of skin involvement and elevated NTproBNP concentrations, respectively, which both displayed an inverse association with all three biomarkers of Se status. The findings are in line with the positive effects of supplemental coenzyme Q10 and Se in reducing NTproBNP concentrations in cardiac patients [45]. Collectively, our analysis supports the classification of SSC as a severe inflammatory disease in which the Se status declines with disease severity [44]. This notion supports the initial hypothesis of our study. However, the data are in some disagreement to a recent report on increased SELENOP concentrations in patients with PAH and a potential involvement of SELENOP in PAH disease etiology [46,47].

An underlying inflammatory nature of SSc as a progressive disease is undisputed, and recent data indicate that pro-inflammatory cytokines are involved in the development of PAH [44,48], such as interleukin-6 (IL-6) [49]. Inflammation appears to synergize with chronic hypoxia in the irreversible induction of pulmonary vascular remodeling and PAH development [50]. Inflammatory cytokines are also known to negatively affect Se status and particularly hepatic SELENOP biosynthesis [51], and molecular studies have highlighted a strong effect of IL-6 and hypoxia on liver Se metabolism and the pattern of selenoproteins expressed under inflammatory conditions [34,52]. Experimental studies in an avian model of PAH indicated that supplemental Se may confer some positive health effects by preventing disease incidence and mitigating PAH symptoms [53]. Hence, it was unexpected to learn that SELENOP levels may be elevated in PAH [47], in view of the ongoing underlying inflammation. This report was also in disagreement with a body of literature describing reduced Se status in SSc [54,55,56]. Moreover, the SELENOP concentrations reported in the PAH patients were outside physiologically reasonable limits, and not compatible with human life [46]. The data presented in this manuscript accord with the skepticism towards the hypothesis on a causal involvement of SELENOP in PAH progression and its potential role as a pharmacological target.

Notably, our results not only disagree with an increased expression of SELENOP in PAH, they rather indicate an inverse relation, i.e., decreasing SELENOP levels with aggravated disease states and a potential loss of SELENOP-related protection against oxidative stress in SSc that may contribute to PAH progression [57]. This interpretation is supported by the tendency of a more severe Se deficit in SSc-PAH as compared to SSc, and by the significant relation to skin involvement or the concentrations of NTproBNP, both known to be associated with SSc severity and survival in PAH [58]. This notion is further supported by prior studies reporting a Se deficit in SSc [56], the inverse relation between Se and NTproBNP [45] and an inverse relation of Se-dependent GPx3 activity with skin involvement and disease severity in SSc [55,57]. The elevated serum CP concentrations observed in SSc are also indicative of increased inflammation and oxidative stress, as CP is an established positive acute phase reactant related to cardiovascular disease [59]. Its potential role in lung disease development has been shown in a murine model of PAH [60], and elevated concentrations of CP in SSc have been observed as early as 1975 [54]. Besides the different markers of Se and Cu status, our analysis also tested the hypothesis that protein biomarkers and composite TE indices may be of particular value and provide higher sensitivity, as recently shown for neonatal infections [41]. While serum Cu concentrations alone were not different between the groups, the Cu/Zn ratio was discriminating between the groups of HC and SSc-PAH. The most pronounced differences between all three groups studied were noted for the ratios of Cu/Se and CP/SELENOP, where even within the group of SSc patients clear differences were present in relation to PAH or NTproBNP. This finding was not totally unexpected given the inverse regulation of serum Se and SELENOP versus Cu and CP under inflammatory conditions, where Se status declines and Cu status increases in blood.

Among the particular strengths of our study are the parallel analysis of several biomarkers of Se and Cu status along with the essential trace element Zn, and the consequent usage of validated methods that yield congruent and plausible results. All three biomarkers of Se status as well as the two biomarkers of Cu status accorded well, indicative of a high quality of the samples analysed and the techniques used. The additional tests for composite biomarkers support their potential value as more sensitive diagnostic readouts, especially when inversely regulated parameters are used, like calculating the ratio of biomarkers of the Se and Cu status in inflammatory settings. Among the limitations of this study are the moderate size of the study groups, and the fact that all of the PAH patients analysed suffer from underlying SSc. Extrapolations to PAH in general are therefore not fully justified, and an analysis of TE status in the different clinical PAH subtypes still needs to be conducted. On the other hand, SSc is a prototypic disease for states of fibrosis, vasculopathy and hypoxia, i.e., a valuable surrogate for latent or early stages of PAH, and the direct comparison between HC, SSc and SSc-related PAH can be viewed as a setup to model disease progression.

## 5. Conclusions

Patients suffering from SSc-PAH display characteristic alterations in biomarkers of their serum Se and Cu status, in agreement with other inflammatory diseases. The Se deficit in patients with skin involvement merits further research as it indicates a potential shortage of protective selenoenzymes needed for an efficient control of oxidative stress and deceleration of scleroderma pathogenesis. An assessment of the composite TE biomarkers Cu/Se ratio and CP/SELENOP ratio, respectively, yielded two novel sensitive and promising diagnostic parameters of SSc and SSc-related PAH, that may enable an early and reliable identification of disease activity and PAH risk. Future studies may test whether supplemental Se and improved selenoprotein expression diminish oxidative stress and positively decelerate disease progression, as indicated in some model systems and clinical studies.

## Figures and Tables

**Table 1:** Characterization of the study cohorts.

**Healthy Controls (HC)**	***n* = 30**
sex, female/male [n/n]	19/11
age, median (range) [y]	53 (23–60)
**Characterization of SSc Patients**	***n* = 66**
sex, female/male [n/n]	49/17
age, median (range) [y]	65 (43–83)
BMI, median (range)	24 (19–48)
Raynaud’s phenomenon, n (%)	61 (92%)
disease duration, median (range) [m]	73 (3–588)
**Auto-Antibodies (abs), Positive Subjects**	
antinuclear abs (ANA), n (%)	66 (100%)
anti-topoisomerase 1 (Scl-70) abs, n (%)	14 (21%)
anti-centromere abs, n (%)	32 (48%)
anti-RNA polymerase III abs (ARA), n (%)	5 (8%)
**Cutaneous Form**	
limited, n (%)	44 (67%)
diffuse, n (%)	18 (27%)
sine scleroderma, n (%)	4 (6%)
**Skin Involvement (*n* = 64/66 data sets)**	**53 (83%)**
mRSS *, median (range)	6 (0–31)
**Pulmonary & Cardiac Involvement**	
pulmonary fibrosis, n (%)	22 (33%)
PAH, n (%)	25 (38%)
NT-proBNP ** [ng/L], median (range)	311 (29–19066)

* mRSS: modified Rodnan skin score; ** NT-proBNP: N-terminal pro-B-type natriuretic peptide.

## Supplementary Materials

The following are available online at https://www.mdpi.com/2072-6643/12/6/1894/s1, Table S1: Prevalence of Se status below (<RefR) or above reference range (>RefR) in SSc patients and HC.
