# Supplementary material for: Selenium and Copper as Biomarkers for Pulmonary Arterial Hypertension in Systemic Sclerosis
Source: Nutrients. 2020 Jun 25;12(6):1894. doi: 10.3390/nu12061894 (PMC7353414; doi:10.3390/nu12061894)
Supplement: Supplementary file 1 [file nutrients-12-01894-s001.pdf]

nutrients

MDPI

Article

Selenium and copper as biomarkers for pulmonary arterial hypertension in systemic sclerosis

Qian Sun[1], Julian Hackler[1], Julia Hilger[2], Hans Gluschke[1], Aldina Muric[1], Szandor Simmons[3], Lutz Schomburg[1,*] and Elise Siegert[2,*]

Supplementary Material

Table S1. Prevalence of Se status below (< RefR) or above reference range (> RefR) in SSc patients and HC

	Reference range	< RefR [%]			> RefR [%]		
		SSc-PAH	SSc without PAH	HC	SSc-PAH	SSC without PAH	HC
Se [µg/L]	52.1–125.1 [17]*	16.0	14.6	0.0	0.0	10.0	10.0
SELENOP [mg/L]	2.9–6.1 [17]*	64.0	41.5	10.0	0.0	0.0	0.0
GPx3 activity [U/L]	196-477 [38]**	28.0	19.5	0.0	0.0	2.4	0.0

*Reference ranges for Se and SELENOP according to the 5th-95th percentile of 598 healthy subjects from the European prospective investigation of cancer and nutrition cohort (EPIC) study. **Reference range for GPx3 activity according to a cross-sectional study of a healthy population (n = 287) from the province of Valencia, Spain.

References

17. Hughes, D.J.; Fedirko, V.; Jenab, M.; Schomburg, L.; Meplan, C.; Freisling, H.; Bueno-de-Mesquita, H.B.; Hybsier, S.; Becker, N.P.; Czuban, M., et al. Selenium status is associated with colorectal cancer risk in the european prospective investigation of cancer and nutrition cohort. Int J Cancer 2015, 136, 1149-1161.

38. Alegria, A.; Barbera, R.; Clemente, G.; Farre, R.; Garcia, M.J.; Lagarda, M.J. Selenium and glutathione peroxidase reference values in whole blood and plasma of a reference population living in valencia, spain. J Trace Elem Med Biol 1996, 10, 223-228.

© 2020 by the authors. Submitted for possible open access publication under the terms and conditions of the Creative Commons Attribution (CC BY) license (http://creativecommons.org/licenses/by/4.0/).
